# Identifying modifiable causes of stress in clinicians and administrators working in New South Wales psychiatric emergency care centres, 2023–24: a qualitative study

**DOI:** 10.5694/mja2.70009

**Published:** 2025-07-24

**Authors:** Jacqueline P Huber, Alyssa Milton, Matthew Brewer, Kat Fry, Sean Evans, Jason Coulthard, Nicholas Glozier

**Affiliations:** ^1^ St Vincent’s Hospital Sydney Sydney NSW; ^2^ The University of Sydney Sydney, NSW; ^3^ ARC Centre of Excellence for Children and Families over the Life Course Sydney NSW

**Keywords:** Emergency services, psychiatric, Suicide, Mental health services, Psychotherapy

## Abstract

**Objectives:**

To examine the experiences of people in various disciplines working in New South Wales psychiatric emergency care centres (PECCs) to identify factors that influence their wellbeing and how they are managed.

**Study design:**

Qualitative study; semi‐structured interviews.

**Setting:**

Eleven of twelve New South Wales PECCs, NSW Health.

**Participants:**

Thirty‐five nurses, psychiatrists, psychiatry registrars, social workers, occupational therapists, and NSW Health staff working in or with management oversight of PECCs.

**Main outcome measures:**

Experiential relativist framework analysis of the experiences of people working in PECCs.

**Results:**

The overarching finding was that working in PECCs involved conflicting, concurrent demands. Three major themes were identified: interactions with the patient: relational work is both meaningful and difficult; interactions with the health care system: conflicting expectations regarding the management of suicide risk causes overwhelming anxiety; and interactions with the hospital: the hospital asks for whatever it needs in the moment, causing clinicians to feel that it does not understand the PECC care model. Two protective factors and processes were also identified: a clear treatment framework reduces uncertainty, enhancing clinician satisfaction and continuity of care for patients; and working in a collaborative team with a flat hierarchy enhances satisfaction and autonomy.

**Conclusion:**

People working in PECCs experience tension and, at times, considerable anxiety arising not just from the intense emotional demands of crisis care but also from the conflicting demands and expectations of the system in which they work. This tension reduces staff wellbeing and retention, and consequently patient care. These negative effects can be reduced by team cohesion and having a clear treatment framework.

**The known**: Australian emergency psychiatry units are a mainstay of inpatient treatment for people at risk of suicide. However, nothing is known about the experience of the clinicians working in these wards.

**The new**: Emergency psychiatry units are subject to conflicting demands related to resource allocation and expectations regarding the management of suicide risk. This tension causes anxiety that can be alleviated by a flat team hierarchy and clear treatment protocols.

**The implications**: Defined care models and effective, evidence‐based inpatient treatment frameworks for people at risk of suicide are essential for the wellbeing of people working in emergency psychiatry units.

Given the international shortage of mental health clinicians^1^, ^2^ and rising levels of compassion fatigue among acute mental health services staff,^3^ reflected locally by the mass resignation of hospital psychiatrists in New South Wales,^4^ understanding the experiences of mental health clinicians in acute care is important. In Australia, psychiatric emergency care centres (PECCs) assess and provide short term treatment for people in crisis, particularly those at risk of suicide. Designed for people with “low to medium acuity mental health problems” and “who are likely to require only brief (up to 48 hours) period of time in hospital”,^5^ PECCs were introduced to improve collaboration between psychiatric and emergency services and to be collocated with emergency departments (EDs). The services themselves, however, were not standardised, and triage processes and service provision within EDs vary according to local requirements.^5^ Bed numbers, governance, and length of stay also vary between centres.

A PECC requires a stable team of experienced staff, but we know little about the factors that influence staff wellbeing.^6^ The experiences of psychiatric nurses have been extensively investigated,^7^, ^8^, ^9^ including with respect to patient coercion^10^ and restraint (felt to be unpleasant but necessary^11^), compassion fatigue^9^ (mitigated by strong leadership, positive workplace culture, clinical supervision, reflection, self‐care, and personal wellbeing), and the relationship of anger with the perceived acceptability of but not the degree of coercion^8^ and aggression (mental health nurses experience more abuse than other nurses).^12^ Less is known about other acute mental health care professionals. Psychiatric trainees for whom psychiatry was not the first choice of specialty are more likely to report burnout,^13^ but enforcing work hour limits, supervision, and stress management tools reduce the risk;^14^ positive work experiences require high quality supervision, supported autonomy, and witnessing patient recovery.^15^

Managing people at risk of suicide can evoke uncomfortable emotions,^16^ intense countertransference reactions such as anxiety, confusion, and anger (in psychiatry residents)^17^ and rejection and inadequacy (in psychiatric nurses).^18^ As individuals experiencing suicidality comprise most of the people seen in emergency psychiatric units such as PECCs, it is possible that the experiences of PECC staff may be different to that of clinicians in other care settings. Research into clinician experiences in this regard has been limited to specialised borderline personality disorder units in the United States and the Netherlands;^19^, ^20^, ^21^ none has been undertaken in Australian emergency psychiatric inpatient units. Information about the clinician experience is required for developing operational frameworks for these complex, often distressing environments.

We therefore examined the experiences of people in various disciplines working in New South Wales PECCs to identify factors that influence their wellbeing and how they are managed.

## Methods

The study reported in this article is part of a broader qualitative study that is evaluating care models and staff practices in New South Wales PECCs.^22^ We report our study according to the Consolidated criteria for reporting qualitative research checklist (COREQ).^23^

### Setting and participants

The study was undertaken in eleven of the twelve New South Wales PECCs: Blacktown Hospital, Calvary Mater Hospital (Newcastle), Campbelltown Hospital, Liverpool Hospital, Nepean Hospital (Penrith), Prince of Wales Hospital, Royal North Shore Hospital, Shellharbour Hospital, St George Hospital, St Vincent’s Hospital Sydney, and Wollongong Hospital (Box 1); the non‐participating unit did not respond to our enquiries. We also interviewed two NSW Health administrators. The clinical director of each unit, as the designated principal investigator, identified potential participants in the unit; purposive sampling (maximum four people per unit) was used to obtain multidisciplinary representation across the eleven units. Nurses, social workers, psychiatrists, psychiatry registrars, hospital managers, and NSW Health staff were eligible to participate if they were working in or had management oversight of PECCs. Further details about the participants and their relationships with the investigators are included in the Supporting Information, part 1.

Box 1 **Work arrangements for the eleven New South Wales psychiatric emergency care centres (PECCs) that participated in our study**

**Table:** 

**Centre**	**Emergency department (ED) coverage by psychiatric emergency care centre team**
1	8.30 am–5 pm: the PECC and ED are both covered entirely by the PECC team (consultant, registrar, two clinical nurse consultants, social worker, junior medical officers). The PECC team also provides an assessment and treatment service to a psychiatric, drug, alcohol, and non‐prescription drug overdose ward attached to the PECC and ED. 5–10 pm: on‐site hospital registrar, clinical nurse consultant for the ED. Psychiatrically trained registered nurses governed by PECC are based in the ED.
2	8.30 am–5 pm: PECC consultant covers the ED; registrar does not. Psychiatric services for the ED are provided by clinical nurse consultants, who do not cover the PECC. After 5 pm: clinical nurse consultants in the ED, on‐call hospital registrar.
3	8.30 am–5 pm: PECC consultant and registrar cover both the ED and PECC. One psychiatric clinical nurse consultant covers the ED only until 10 pm; after 10 pm: on‐site hospital registrar.
4	8.30 am–5 pm: PECC consultant covers the ED; ED is also covered by two clinical nurse consultants and an unaccredited registrar from another hospital. According to demand, the PECC registrar can be called on to cover the ED. After 5 pm: on‐site hospital registrar.
5	8.30 am–5 pm: PECC consultant available solely for phone calls from clinicians covering the ED; PECC registrar covers ED in person. After 5 pm: on‐site hospital registrar.
6	8.30 am–5 pm: the ED is covered by two clinical nurse consultants, and two non‐PECC registrars who also cover the crisis team; career medical officer covers the PECC; all can be sent to the PECC if required. After 5 pm: clinical nurse consultants for the ED (until 10 pm) and on‐site hospital registrar.
7	Mental health‐specific ED; general ED staffed by psychiatric registrars and nurses; PECC team does not cover the ED. After 5 pm: on‐site hospital registrar.
8	8.30 am–5 pm: both the ED and PECC are covered by the PECC consultant and registrar; ED also has an ED‐only psychiatric clinical nurse consultant. After 5 pm: on‐site hospital registrar.
9	PECC is a mental health ED, with access to four short stay emergency mental health inpatient beds. Patients are triaged in the attached general ED and transferred to the PECC, where they are tended by the PECC team in the same way as an ED tends people with non‐psychiatric problems. The PECC team can also perform assessments in the general ED. PECC is available 24 hours a day.
10	8.30 am–5 pm: The ED is covered by several mental health clinical nurse consultants and the PECC consultant, and the PECC registrar for one hour each morning and then a dedicated ED psychiatry registrar. After 5–10 pm: Clinical nurse consultants and on‐site hospital registrar covers ED.
11	This unit moved from the ED to a separate area about two years ago. Instead of the PECC, services in the ED are provided by clinical nurse consultants and clinical nurse specialists and a daily roster of registrars; psychiatrists provide an on‐call phone service at all times.

### Data collection

During 22 June 2023 – 20 February 2024, semi‐structured interviews using questions developed by authors JH, NG, and AM (Supporting Information, part 2) were undertaken, with one or two participants at a time. We aimed not for data saturation but to comprehensively examine staff practices in all PECCs by purposively sampling all participating units. After each participant provided informed consent in an online form and received information about the study from author JH (or AM, at JH’s clinical workplace, St Vincent’s Hospital), semi‐structured interviews (mean duration, 50 minutes) were conducted by a PECC psychiatrist (JH) or psychologist (AM, at JH’s workplace). Participants were not financially compensated. The interviews were transcribed using the transcription feature of Microsoft Teams, and the data were cleaned and anonymised by JH.

### Analysis

We analysed the interview transcripts thematically using a semantic and latent focus^24^ and an experiential relativist framework. Data were analysed iteratively by a psychiatrist (author JH), peer worker (KF), Aboriginal health worker (JC), clinical nurse consultant (MB), and senior manager/nurse (SE). First, JH familiarised herself with all transcripts; KF, JC, MB, and SE each familiarised themselves with up to four transcripts. Each reader then generated initial codes, followed by a series of meetings, in which codes and then initial themes were created using memos, a cork board, and paper. JH coded all transcripts and each other analyst coded one to three transcripts in NVivo 14, aiming for deeper understanding and assessing code appropriateness. All analysts participated in a codebook and further theme development. The themes were refined in a reflexive process and iteratively discussed with authors AM and NG as they were identified. All transcripts were coded and checked, and ten were each coded by two analysts. A report of the results was prepared by the team and a lay summary for the participants. Information about the investigators, including their reflexivity statement, is included in the Supporting Information, part 1.

### Ethics approval

The St Vincent’s Human Research and Ethics Committee approved the study (2022/PID00456).

## Results

We interviewed 35 people from eleven units and at NSW Health (two to four per unit; nineteen men, sixteen women): eleven senior nurses (including eight administrators: five with and three without clinical responsibilities), nine psychiatrists (including five administrators: four with and one without clinical responsibilities), seven psychiatry registrars, three junior nurses, two social workers, one occupational therapist (an administrator without clinical responsibilities), one hospital administrator without clinical training, and two NSW Health staff administrators (one with, one without clinical training; neither with clinical responsibilities). The interviewees included nine hospital managers with clinical responsibility (four clinical directors, five nurse unit managers) and five without clinical responsibility. Psychiatry work experience duration ranged from two weeks to 35 years, and interviewees had up to ten years’ non‐continuous experience in PECCs.

The overarching finding was that working in PECCs involved conflicting and concurrent demands (Box 2). We identified three areas of dissonance: working with people at risk of suicide and their families and carers led to intense, exhausting, and sometimes intolerable emotions, but also connection and a sense of reward; conflicting system expectations that suicide risk must be contained by admitting people to hospital and that, as the risk cannot be contained, people should be discharged from hospital; and PECCs should both provide long stay admissions and function as short stay units.

Clinicians reported that these tensions often led to unmanageable anxiety that could leave them feeling uncertain and not having control without two protective factors: clearly defined treatment frameworks that either acknowledged or helped manage these tensions; and support from the collaborative, flat hierarchical team structure in their units, which enhanced control and autonomy.

Box 2 **The conflicting organisational and care system demands associated with working in New South Wales psychiatric emergency care centres: summary of findings from 35 interviews, 22 June 2023 – 20 February 2024**

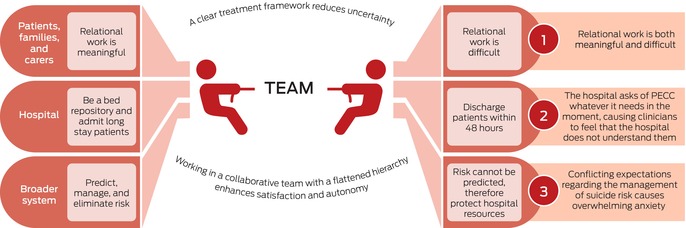

### Interactions with the patient: relational work is both meaningful and difficult

Participants simultaneously experienced satisfaction and intensely uncomfortable emotions. The more pleasant emotional experiences involved human connectedness and feedback about a job well done. However, these feelings were coupled with strong emotions, as frequently and passionately raised by participants. This was a gruelling daily experience that senior staff members believed harmed junior staff, exacerbated by feeling overwhelmed by the number of people clinicians are expected to manage. Interestingly, specific emotions were not labelled; instead, clinicians described the amount or effect of the emotion, such as “exhausting” and “intense” (participants 8 and 9) or “transference is just very, very strong” (participant 1), possibly because they do not feel free to discuss specific unpleasant emotions related to patients. Staff described doing their best to manage, but this was reported neither confidently nor often (Box 3).

Box 3 **Interactions with the patient: relational work is both meaningful and difficult. Illustrative quotes**

**Table:** 

You’re a human being and you’re treating them as a human being and you kind of understand them and figure out what they need. (participant 4, registrar)
So and definitely one of the other things is a really great feedback from our patients in regards to our staff, the way they clinically practice that makes us, gives us satisfaction. (participant 5, senior nurse)
The, the emotional intensity of the highly traumatised patients that end up in PECC has been, I think, the main issue that people have, you know, wanted to go and do something a bit more straightforward like treat people with schizophrenia. (participant 6, psychiatrist/administrator)
Everyone has their biases, so it can be personally can be tiring for me to be able to deal with, I guess, people that have some sort of like or personality vulnerabilities like day in, day out sort of thing or like repeated a patients in a day. So I would say that I’m a very empathetic person, but at times it can with like thin. (participant 7, nurse/administrator)
The registrars are kind of exhausted at the end of every six month period … they were, totally almost all of them had, I would have thought, some like adjustment disorder. Honestly, I’m being really frank. (participant 6, psychiatrist/administrator)
You know when I’m working in the mornings, it’s very, very intense. It’s absolutely exhausting, you process vast amounts of information. (participants 8 and 9, nurses)
But I think that helps in the way you can … the way that you work with a person and continue persevering despite those days where it’s really, really tricky with certain patients where that, the transference is just very, very strong there. And you know, we will come up with different ways to manage that. I just, to me, it’s like remembering that. You know, the way that they all see things, the way they do or react to things because of so many things, including their past traumas. (participant 1, social worker)

PECC = psychiatric emergency care centre.

### Interactions with the health care system: conflicting expectations regarding the management of suicide risk causes overwhelming anxiety

PECCs were seen as the major focus of suicide risk in inpatient facilities. “Risk” was frequently mentioned, including in the context of risk tolerance, risk containment, and risk ownership. Clinicians acknowledged that “it’s impossible to predict risk” (participant 20) but felt under pressure to measure, manage, and take responsibility for it. Senior nurses described feeling responsible for “taking the risk” of harm for discharged patients (participants 2 and 3), despite being unable to control it. The burden of risk ownership and the anxiety it caused were frequent themes, reflecting the conflict between the reality of unpredictable risk and the expectation of eliminating it (Box 4).

Box 4 **Interactions with the health care system: conflicting expectations regarding the management of suicide risk causes overwhelming anxiety. Illustrative quotes**

**Table:** 

I think, you know, a lot of those patients, you know their behaviours are so confronting and scary for a service and for staff that the push can be to just become more and more coercive with the things that you do and more and more restrictive, which has terrible outcomes. So I think part of the kind of medical role is actually just holding people in, and like taking that risk on. (participant 4, registrar)
If they keep saying they’re unsafe, they’re unsafe, they’re unsafe, at some stage you’re gonna have to discharge and even if it’s months, weeks later, you’re going to have to take that risk sooner or later. (participants 2 and 3, nurses)
A big part of that expectation has fallen onto the senior clinician in our team for sure, to kind of resolve a lot of the issues that are happening and, and assumes a lot of risk. And I guess that, that feeling has filtered down, I think, to the, the rest of us, in the sense that I feel like we’re taking a lot of risks with people at the moment. I’m, I’m like you know, again, like it’s one of those things, doing this role there’s always gonna be an element of apprehension anytime that someone’s discharged or whatever you know. But at the moment it feels as if that’s heightened. (participant 13, nurse)
Look, I I think as well it unfortunately it can be personality driven too, you know, and it just doesn’t always suit everybody you know and some people don’t like to wear the risk … with the chronically suicidal, we have to wear that risk at some point, and you’ve got, you know, people that can sit and feel comfortable with that. And then you’ve got others that can’t, and I see that all the time in terms of people coming through the door, you could almost tell who’s been on call overnight. (participant 14, nurse/administrator)
It’s difficult to retain people in the public sector, but PECC has been particularly difficult. And I think one reason is that you do tend to attract a little cohort of coroner’s cases after a while, and that becomes very burdensome and frightening for some people. I know that’s why one of our longest, longest standing, most dedicated sort of PECC consultants left … I had a guy recently leave pretty much because he began to feel increasingly exposed after a series of suicides … and even single suicides have knocked some of those clinicians out. And they just haven’t recovered from it.
Part of the reason I’m here, I can’t remember the exact number but it’s certainly more than ten … I think it’s about fourteen consultants in the PECC in as many years. I mean that’s how they operate. They burn through people … Our average length of occupation in an ED PECC position is one year. (participant 6, psychiatrist/administrator)

PECC = psychiatric emergency care centre; ED = emergency department.

The knowledge that suicide risk is unpredictable was associated with justification of protecting limited system resources. Participants noted the expectation for clinicians to make “risky” decisions without a clear framework for doing so, and not making these decisions being viewed as an abnegation of responsibility. A senior nurse described how external system pressure on their lead clinician to take risks led to team‐wide “apprehension”. Some participants (clinicians and administrators) reported that clinicians with conservative approaches to risk are seen less favourably than those who can tolerate the anxiety of discharging a patient at risk of harm (Box 4).

Clinicians perceived the dual expectation to ensure safe care while making risky decisions to conserve resources. It was suggested that the anxiety caused by these conflicting expectations contributed to poor retention of psychiatrists. Clinical directors noted that the psychiatrists bear a heavy burden with respect to suicides because of anxiety‐provoking scrutiny, particularly from the coroner (Box 4).

### Interactions with the hospital: the hospital asks for whatever it needs in the moment, causing clinicians to feel that it does not understand the PECC care model

PECCs managed patients with complex biopsychosocial problems, the “Swiss army knife of psychiatry” (participant 6), which the system does not see or understand, as recognition of this complexity has faded (Box 5).

Box 5 **Interactions with the hospital: the hospital asks for whatever it needs in the moment, causing clinicians to feel that it does not understand the PECC care model. Illustrative quotes**

**Table:** 

No one really arrives in PECC just because they have a mental disorder. They have a mental disorder, poor social support, substance abuse and, you know, suicidality, which is I think quite a different dimension to having a mental disorder. (participant 6, psychiatrist/administrator)
It always used to be when I first started that the most experienced registrar would be the PECC registrar. That’s not the case any more. (participant 20, psychiatrist)
It does sort of feel like they’re just churning through patients here because they feel they can do it quickly. And regardless of the actual outcomes for the patient, is the bed empty? How many people we’ve had through in a week, we also get thrown in our faces by the bed manager. You’ve gone over the 48 hour mark. You know, that’s not good enough. (participants 2 and 3, nurses)
The bed manager you know, there’s a lot of patients in the emergency department and there’s a bed in the PECC unit. Sometimes patients are who are not meant to be in PECC end up there. Uh, so which is not supposed to be? Yeah, even psychotic patients. Some of you find them … Yeah, in the PECC ward. And they’ll breach the 72 hours they’ll be there for a long time. And because there’s no beds in long stay units and also patients who are homeless can be challenging uh to discharge because of accommodation issues. (participants 15 and 16, nurses)
We had to shut it down because there was work being done on the [undisclosed] unit and they didn’t realise. They just thought “oh no, it’s just a four bed, you’ll be fine” when, hang on, these four beds turn over more people in a day than an entire acute unit does. (participant 17, occupational therapist/administrator)
Because their view was that if the beds were occupied close to 100%, that means everyone was doing a good job. And, and I said to them, listen, we’ve got four beds, if we’ve got anything more than 75% occupancy, that means when you want to get someone in, you can’t. Umm. I said I, I’m aiming for 50% occupancy, which of course might cause them conniptions. I said … because at 50% occupancy then we that that gives me wiggle room on a daily basis. But if you, if you’re gonna be obsessed with the idea of pushing it up towards 100%, then you’re not gonna have the flexibility in the system which you thought was gonna be one of the benefits of this of this model. (participant 18, psychiatrist/administrator)
How do you safely discharge someone when you’ve not been able to offer any change? (participants 2 and 3, nurses)
We always need to make a referral and kind of I feel that sometimes we are asking for a favour or we need to, like, at some points I feel like I’m begging. (participant 12, psychiatrist)
Some would probably be better managed in a primary drug and alcohol facility if the truth be known, but again we don’t have any drug and alcohol beds. Our drug and alcohol beds are in another hospital, and that hospital has no effective psychiatric service. So they’re unable to tolerate any degree of perceived suicide risk. (participant 6, psychiatrist/administrator)
I don’t want to be dealing with management issues. I don’t want to be dealing with politics and bureaucratic shit. Like I’d just like to go in and assess the patient … The biggest thing the primary clinical staff hate the most is pressure from ED and management to get the patient through. And so all the things that come alongside that, you know, the politics and everything … The ideal world? Have all the time and enough resources to just treat the patient. (participant 19, registrar)

PECC = psychiatric emergency care centre; ED = emergency department.

This perceived lack of understanding was related by participants to two diametrically opposing stances. On the one hand, clinicians were under pressure to discharge people within the 48 hour period specified by the NSW Health model of care,^5^ despite the complexity of patient needs and limited unit resources. On the other, most clinicians felt compelled to fill beds with people expected to require longer admissions when ED capacity had been exceeded and the long stay inpatient ward was full; PECCs are used to manage bed numbers rather than as part of a care model. This feeling was noted by interviewees at all seniority levels. Clinicians felt their ability to ease system pressure was undervalued, noting that rapid turnover reduces lengths of stay by providing non‐specialist care, and lower bed occupancy facilitates flexibility and more rapid flow of patients through the ED (Box 5).

Clinicians also felt they did not have the resources to manage the complexity of the patients in their care. Social workers, in particular, felt under pressure to change the social circumstances of individuals despite their limited options, feeling set up to fail by system‐level features beyond their control. Helplessness was felt by staff who could not effect change for people living in distressing circumstances, particularly units without dedicated social workers or drug and alcohol services. This led to perceptions that the system was unjust and fragmented, with few outpatient resources, angering clinicians who felt under pressure to make decisions without the appropriate resources (Box 5).

### Protective factors and processes

#### A clear treatment framework reduces uncertainty

The perceived clarity of the local treatment framework affected the experience of clinicians within the team and their interactions with patients. A clear treatment framework provides a sense of stability and satisfaction for clinicians and of continuity of care for service users. Clinicians who spoke about their treatment frameworks with precision and confidence were more likely to describe enjoyment in their role, a better running system and team, and better patient experience. Conversely, those who reported that the treatment approach was not clear often felt unsure of themselves and their clinical efficacy, felt that their scope of practice was poorly defined, and were frustrated in their ability to effect change. This feeling was expressed at all seniority levels (Box 6).

Box 6 **A clear treatment framework reduces uncertainty. Illustrative quotes**

**Table:** 

Because I really like, you know, [name] notwithstanding, I really felt the love at [hospital] … and the registrars I was surprised to hear, strongly agree. And despite the myriad problems we experience, it’s the love that they feel. So that’s. Kind of. It’s a great working relationship … I think a lot of people were scared to admit to PECC without a plan … but now they’re actually trying to make up plans. I think it’s had two great roll on effects. I think the admission plans to the acute wards are getting better as well. But also once you work out a plan, sometimes you [can] discharge the patient because once you tell the patient the plan, they can just do it in one day and go, yeah, so … crisis admissions are being better managed overall because there is this ethos of thinking, “this is how you handle it”. (participant 10, psychiatrist)
Even though it’s a line in my job description but in [health provision location], [job type] are case managers, we are not counsellors. “That’s why we have psychologists [name].” (participant 11, social worker)
Ideally it would be great to have at least kind of more permanent staffing regarding a psychologist because it’s … we had a patient who presented with some complex, complex trauma and interpersonal difficulties, like lots and lots of stresses that needed to be addressed by a psychologist because you know, it’s actually, this was destabilising, the patient was having suicidal ideation. But the psychologist was only able to see the patient once on the second day of the admission, and then on discharge two weeks later” (participant 12, psychiatrist)

PECC = psychiatric emergency care centre.

#### Working in a collaborative team with a flat hierarchy enhances satisfaction and autonomy

Comments about job satisfaction or enjoyment were often related to team function. Most teams were described by their staff as highly collaborative, compassionate and holistic, and interviewees valued their working relationships. Social workers and senior nurses frequently and favourably referred to the flat hierarchy of PECCs, as well as commenting that working in PECCs was more challenging (in a positive sense) than acute inpatient wards. Other positive interprofessional experiences reported by senior nurses and registrars concerned specific senior team members and highlighted the importance of an experienced and calming presence in the ward (Box 7).

Box 7 **Working in a collaborative team with a flat hierarchy enhances satisfaction and autonomy. Illustrative quotes**

**Table:** 

The values, the sort of holistic approaches from like the other staff, especially our clinical nurse consultants, who are amazing. And I think it also balances out with having a social worker on the team as well. And also the fact that they really do take into account my opinions, my suggestions when I do have any that is of any weight. So I think I was lucky enough that they would actually, I mean, I never felt that I was “just a social worker” or anything like that. I felt I was really privileged to have worked with a really close knit team and one that we worked very well together. (participant 1, social worker)
We all make decisions together as a team rather than following what someone said at the top. (participants 2 and 3, nurses)
It’s quite enjoyable, just a little bit more challenging than general nursing roles in the inpatient ward. (participants 2 and 3, nurses)
I was very contained by [supervisor’s name] and kind of working with someone so experienced and be like around each other all the time … we would do interviews in a very collaborative way and usually both of us would be kind of talking at some stage. Some say that was kind of learning a lot through that model of apprenticeship … but a lot of PECCs sort of don’t have that level of consultant cover, so … (participant 4, registrar)

PECC = psychiatric emergency care centre.

## Discussion

We report the first study to qualitatively investigate the experiences of clinicians working in emergency psychiatric units (PECCs), undertaken by an investigator team with varied skills and perspectives. Our study is also timely, given the unrest among psychiatrists in the New South Wales public health system. Overall, we found that clinicians view PECCs as characterised by co‐occurring, opposing pressures that reflect both resource limitations and conflicting cultural views of risk.

### Inadequate connections between care system groups cause problems with understanding other people in the system

We found a clear divide between “us” (the team) and “them” (the hospital and health system). This divide is unsurprising, given the pressure of the PECC environment, where conflicting priorities and intense emotions are common. Stressful environments often lead to close knit, polarised groups, with both positive and negative consequences.^25^ They enable staff to manage emotional demands, but people in polarised groups often have trouble understanding people from outside their own group. The adaptive mentalisation‐based integrative treatment (AMBIT) framework emphasises the importance of connections with the broader system without which “a tightly interconnected … team is condemned to … [relying] on a pretence of self‐sufficiency.”^25^ These connectors must be epistemically trustworthy; that is, they must be trusted to provide relevant, generalisable information. Establishing connectors in PECCs is complicated by complex system interfaces, but epistemic trust between clinicians and management is imperative for managing the tensions between care model fidelity, expectations about managing risk, and limited hospital resources. Constant mentalising (understanding one’s own and others’ mental states through thoughts, feelings, and intentions) between teams is hard work, and requires a relational framework for this purpose.

### To relieve the tension associated with managing suicide risk, let us instead concentrate on minimising suffering

Clinicians report that they are expected to predict and manage risk by admitting people as inpatients, but also to discharge people to conserve resources or to limit admissions to accommodate transfers from the ED. This tension is reflected in the literature. Risk prediction and stratification for clinical decision‐making are regarded as futile in psychiatry,^26^ and the evidence that hospitalising people with suicidal ideation prevents suicides is limited.^7^ Nevertheless, in emergency psychiatry the term “risk assessment” is often used synonymously with “clinical assessment”, and the term “zero suicides” has been discussed in the academic literature as fostering blame and inappropriate guilt in clinicians if future harm is not predicted.^27^ The dissonance between clinicians’ knowledge of the evidence for what they do, and the social discussion of suicide, supports their perception of conflicting expectations that provoke anxiety. Given the limited ability to prevent suicide, it might be better to give priority to goals other than identifying suicide risk factors, instead concentrating on elements associated with distress and self‐harm^28^ to alleviate suffering. Clinicians could then avoid the conflicting expectations of managing risk through admission and discharge. Mental health professionals could reduce the tension by incorporating empirical evidence into staff training, educating patients, families, and carers, and ensuring that this evidence is reflected in policy and care models.

### Clinician wellbeing will require action

Clinician wellbeing was a recurrent motif in the interviews. The job demands–resources theory (JDRT), a framework for assessing how psychosocial work factors affect wellbeing,^29^ suggests that conflicting or high work demands without adequate supporting resources leads to distress and burnout, whereas autonomy, control, and collegial and managerial support reduce the risk.

Clinicians in PECCs report experiences that can be directly related to this framework: constant conflicting demands regarding resource use (accepting people for potentially long stays but remaining a short stay unit), conflicting expectations regarding risk management (protecting people at risk of suicide by admitting them but acknowledging that admission does not reduce the suicide risk), and the emotional challenges of the work itself.

It may not be possible to reduce some of these demands in emergency psychiatry. However, we found that the impact of these conflicting demands could be mitigated by a clear treatment framework. This finding illustrates the JDRT buffering effect of enhancing autonomy and control to reduce the job strain caused by high demands and low control. The organisational details need further consideration, but AMBIT and mentalisation frameworks could be applicable, offering both an emergency psychiatry treatment framework^30^ and enhancing autonomy and support.^25^ This approach merits future examination for improving clinician wellbeing, retention, and patient care. The participants also reported collegiality arising from collaborative, flat hierarchy teams, but this finding could reflect recruitment bias in our study. Reducing staff turnover and increasing managerial and team support^31^ could improve the staff experience.

The recent mass resignation of psychiatrists from the New South Wales public health system,^4^ after this study was completed, indicates that system improvement is important for clinician wellbeing. In drawing attention to an ailing system, the resignations have probably exacerbated the sense of conflicting expectations among remaining clinicians. The pressure on PECCs to perform outside their intended model of care may increase, and the staff experience deteriorate further. While a clear treatment framework and clarity about expectations regarding risk management are imperative, adequate funding and staffing and must come first, “organised around a mentalised understanding of the needs and wishes of the client rather than the requirements of the specific agency in which a worker is employed.”^32^

### Future directions for research and policy

Organisational interventions to enhance clinician and manager wellbeing, rather than relying on individual approaches, are available but are infrequently implemented and rarely evaluated. A recent review found that fewer than 10% of interventions for enhancing clinician wellbeing were organisationally focused.^33^ Frameworks to design and evaluate such interventions are available^31^ and have been embedded in legal regulations.^34^ We identified key targets for developing and testing organisational approaches, including aligning suicide risk assessment and prevention with published evidence, reconsidering treatment goals, and reducing conflicting organisational demands.

The important and sometimes fraught relationships between emergency psychiatry and ED clinicians should be investigated further, as well as the experience of service users and their families and carers. In particular, we need evidence about situations in which there are disagreements — within families and care networks, or between families and care networks, service users, and clinicians — regarding treatment decisions, including the use of detention under the *Mental Health Act*.

### Limitations

We aimed for full representation of PECC clinicians in New South Wales, but emergency psychiatry units in other Australian states may have different perspectives to those we have reported. The sample size was kept small to facilitate intensive qualitative analysis, but the generalisability of our findings is unknown. We do not know how many people declined to participate in the study. All authors are health researchers, narrowing the analytical focus.

## Conclusion

People working in PECCs experience tension and, at times, considerable anxiety arising not just from the intense emotional demands of crisis care but also from the conflicting demands and expectations of the system in which they work. This tension reduces staff wellbeing and retention, and consequently patient care. These negative effects can be reduced by team cohesion and having a clear treatment framework. Our findings could help identify and evaluate organisational approaches to improving the wellbeing of people in these demanding roles.

## Open access

Open access publishing facilitated by The University of Sydney, as part of the Wiley – The University of Sydney agreement via the Council of Australian University Librarians.

[Correction added on 22 August 2025, after first online publication: CAUL funding statement has been added.]

## Competing interests

No relevant disclosures.

## Data sharing

The data for this study will not be shared, as we do not have permission from the participants or ethics approval to do so.

## Author contributions

Jacqueline P. Huber: conceptualisation, data curation, formal analysis, investigation, methodology, project administration, writing (original draft, review and editing), funding acquisition. Alyssa Milton: conceptualisation, data curation, investigation, methodology, project administration, supervision, writing (review and editing). Matthew Brewer: conceptualisation, formal analysis, writing (review and editing). Kat Fry: conceptualisation, formal analysis, writing (review and editing). Jason Coulthard: conceptualisation, formal analysis, writing (review and editing). Sean Evans: conceptualisation, formal analysis, writing (review and editing). Nicholas Glozier: conceptualisation, data curation, investigation, methodology, project administration, supervision, writing (review and editing).

Received 16 October 2024, accepted 10 March 2025.

## Supporting information


**Data S1:** Supplementary methods
