# Supplementary material for: Identifying modifiable causes of stress in clinicians and administrators working in New South Wales psychiatric emergency care centres, 2023–24: a qualitative study
Source: Med J Aust. 2025 Jul 24;223(8):410–7. doi: 10.5694/mja2.70009 (PMC12536082; doi:10.5694/mja2.70009)
Supplement: Supplementary file 1 — Data S1: Supplementary methods [file MJA2-223-410-s001.pdf]

MJA
The Medical Journal of Australia

Supporting Information

Supplementary methods and results

This appendix was part of the submitted manuscript and has been peer reviewed. It is posted as supplied by the authors.

Appendix to: Huber JP, Milton A, Brewer M, et al. Identifying modifiable causes of stress in clinicians and administrators working in New South Wales psychiatric emergency care centres, 2023–24: a qualitative study. *Med J Aust* 2025; doi: 10.5694/mja2.70009.

Supplementary methods

1. Supplementary method details

Design:

We undertook a qualitative analysis, taking an experimental relativist approach, with the goal of sampling every Psychiatric Emergency Care Center (PECC) in NSW, as well as NSW Health staff.

Between 1 February 2022 and 14 April 2022, a semi-structured interview schedule was designed by Jacqueline Huber, Nick Glozier and Alyssa Milton (section 2). The questions meant to elicit responses to do with PECC models of care (as part of a broader study), as well as the experiences of staff working on PECCs (the focus of this study). Questions were designed to be open, with closed question prompts if open questioning yielded small amounts of information. The interview aimed to ask one question at a time, with neutral wording.

Once the interview schedule was written, Jacqueline Huber approached Matthew Brewer, Kat Fry, Jason Coulthard and Sean Evans to join the research team, review the interview schedule, and discuss the analysis method and framework.

Inclusion criteria:

Inclusion criteria pertained to those working on, or had management oversight of, their local PECC, and included nurses, social workers, psychiatrists, psychiatry registrars, hospital managers, and NSW Health staff.

Setting:

The setting was 11 of 12 New South Wales (Australia) PECC units: Blacktown Hospital, Calvary Mater Hospital, Campbelltown Hospital, Liverpool Hospital, Nepean Hospital, Prince of Wales Hospital, Royal North Shore Hospital, Shellharbour Hospital, St George Hospital, St Vincent's Hospital Sydney, The Wollongong Hospital of 12 NSW PECCs and 1 Ministry of Health site). Researchers approached several staff affiliated with the 12th PECC, via email, but did not elicit a response.

Participants and recruitment:

Recruitment

Each site's principle investigator was the local clinical director. He or she identified potential participants that satisfied inclusion criteria, with purposive sampling aiming to obtain multidisciplinary representation across sites. Participants were working on, or had management oversight of, their local PECC, and included nurses, social workers, psychiatrists, psychiatry registrars, hospital managers, and NSW Health staff.

An email proforma was provided by the research team to principal investigators. Principle investigator emailed potential participants who met eligibility criteria and gained their consent to refer them on to the researcher.

Consent

The researcher (either Jacqueline Huber or Alyssa Milton, and Jacqueline Huber's place of work) made email contact in order to explain the study and obtain consent. Prior to interview, participants had the opportunity to review the participant information and consent forms, and discuss any questions, before giving informed consent. The interviewer made an appointment for the interview via email after consent was acquired.

Relationships between researchers and participants

Four participants had professional relationship with Jacqueline Huber, but the participants and Jacqueline Huber had not specifically discussed this project prior to the consent process. Co-authors only reviewed de-identified data thus had no personal relationship with, or knowledge of participants. Participants were informed about who the involved researchers were in the participant information statement.

Data collection

Semi-structured interviews were conducted by PECC psychiatrist (Jacqueline Huber) or a psychologist (Alyssa Milton, at Jacqueline Huber's place of work) and averaged 50 minutes in duration. Participants were not financially compensated. Interviews were transcribed using the 'transcription' feature of Microsoft Teams, and data were cleaned and anonymized by Jacqueline Huber.

Analysis:

Data were analysed iteratively by a psychiatrist (Jacqueline Huber), peer worker (Kat Fry), Aboriginal health worker (Jason Coulthard), clinical nurse consultant (Matthew Brewer), and senior manager/nurse (Sean Evans). Analysis took the following steps: 1) Jacqueline Huber became familiar with all data; Kat Fry, Jason Coulthard, Matthew Brewer, and Sean Evans familiarized themselves with up to 4 transcripts; 2) each member generated initial codes; 3) over several meetings, first codes and then initial themes were created using memos, a cork board and A3 paper; 4) all analysts coded up to 3 manuscripts, while Jacqueline Huber coded all, in NVivo 14, aiming for deeper understanding and assessing code appropriateness. All analysts participated in a code-book and further theme development; 5) themes were refined in detail through a reflexive process and iteratively discussed with Alyssa Milton and Nick Glozier as they were identified, all transcripts were coded and checked, and 27% double-coded. The results were written by the team and a lay summary was returned to participants.

Researchers:

Researcher credentials

Jacqueline Huber: BSc, MBBS, FRANZCP, MMed

Alyssa Milton: BSc, PGDip Psych, MAppSc Health Psych, PhD

Nick Glozier: MA, MBBS, MSc, MRCPsych, FRANZCP, PhD

Sean Evans: Bachelors in Mental Health Nursing, Registered Nurse, Post Graduate Certificate in Health Management and Leadership

Kat Fry: Bachelors of Mass Communication, TAFE Certificate 4 in Mental Health and Certificate 4 in Drugs and Alcohol, Peer Leadership Skillset Training

Jason Coulthard: Bachelor of Social Work, Diploma of Case Management, TAFE Certificate 3 in Aboriginal Primary Health Care

Researcher occupations

Jacqueline Huber: practising psychiatrist in an emergency department and PECC in an inner-city hospital

Alyssa Milton: psychologist and research fellow specializing in qualitative and mixed methods research, and has Australian and international experience working in early psychosis

Matthew Brewer: clinical nurse consultant in an emergency department and PECC in an inner-city hospital

Sean Evans: senior nurse and senior manager of a mental health and drug health service in an inner-city hospital

Kat Fry: peer worker in psychiatric inpatient and community environment in an inner city hospital

Jason Coulthard: Aboriginal mental health worker in an inner-city hospital

Nick Glozier: practising psychiatrist and academic researcher in an inner-city hospital

Researchers' gender

Female: Jacqueline Huber, Alyssa Milton, Kat Fry

Male: Matthew Brewer, Sean Evans, Jason Coulthard, Nick Glozier

Researchers' experience and training

Two authors (Nick Glozier and Alyssa Milton) are active, experienced researchers with expertise in qualitative, quantitative, and mixed methods approaches. One author (Jacqueline Huber) is a PhD candidate. The other authors (Matthew Brewer, Kat Fry and Jason Coulthard) have some research experience. All have clinical experience, ranging from 3-10 years.

Reflexivity statement:

Jacqueline Huber is a practising psychiatrist working in an ED and a PECC in inner city Sydney, completing a PhD, who works within a framework informed by mentalization based therapy, transference focused psychotherapy, and dialectic behavioral therapy. She has interest in emergency psychiatry, and organizational responses to undesirable outcomes in health. Alyssa Milton is a psychologist and research fellow with Australian and international experience. Matthew Brewer is a clinical nurse consultant working towards a nurse practitioner license, with an interest in emergency psychiatry, mentalization based therapy, good psychiatric management, and metabolic health of people with mental illness. Sean Evans an experienced senior nurse and manager with Australian and international experience. Kat Fry is the program peer coordinator at an inner city hospital and local non-government organization, with a particular interest in holistic health methodology. Jason Coulthard is an Aboriginal mental health worker who performs in-reach to inpatient wards, with experience in the case management of youth in inner city Sydney, and an artist. Matthew Brewer, Sean Evans, Kat Fry, Jason Coulthard and Jacqueline Huber work together at the same hospital. Nick Glozier is a practising community psychiatrist at another inner city Sydney hospital, and an academic who specializes in epidemiology, trials, and health science research. Five researchers on this paper work together, in a specific PECC treatment environment, viewing the data through the lens of their existing opinions and experiences. All are practicing health workers, which may provide a deeper understanding of the experience of the participants, while potentially obscuring others. The analyst choice was intended to broaden the available viewpoints to include as many as possible within hospital mental health system.

2. Semi-structured interview questions used in the study

Introduction:

- Thank you for meeting me here today and offering to take part in this study.
- First I'll quickly outline the study and confirm that everyone has signed the consent form
- I have a list of topics I'd like to discuss
- Feel free to ask questions at any time
- I might make some notes as we talk
- Just a reminder that this will be audio-recorded. Can I confirm that's ok?

Topics and questions

Background information:

- Current role and duration of employment
- Identify service model details
  - o How many of each clinician type, and what Full Time Equivalent (FTE)?
  - o Do some of your clinicians cover ED as well, or solely the PECC/Mental Health Short Stay Unit (MHSSU)?
  - o Does the staffing profile of your PECC/MHSSU affect the clinical model of care?
  - o Are you aware whether or not your unit has a Model of Care? If yes, are you able to give me a brief description?

What do you feel are the overall objectives of PECCs? Prompts:

- Are there system objectives?
- Are there clinical objectives?
- Are there political objectives?
- Other objectives that we haven't thought of?

Do you feel there is a difference in expected objectives for a PECC vs an Acute Mental Health Unit? Prompts:

- If yes, in what way?
- If no, what is the purpose of having a PECC separate from the Acute Mental Health Unit, in your opinion?
- Have a different clinical model of care?
- Have a different suite of treatments? If so, what treatments should PECCs have available?

Understand expectations of clinical approach

- What do you feel are the goals of treatment at your PECC/MHSSU?
- Do you think that suicide prevention is a goal?
- Has this changed over time?
- What is your role at your PECC/MHSSU?
- Does your role influence the types of interventions you use?
- What do you believe the hospital management expects of a PECC?
- What do you believe NSW Health expects of a PECC?
- What do you believe consumers expect of a PECC?

Supplementary results

Table 1. Participant characteristics

		Number of participants	Administrator with current clinical responsibility	Administrator without current clinical responsibility
**Participant type**	Psychiatrist	9	4	1
	Registrar	7		
	Senior nurse	11	5	3
	Junior nurse	3		
	Social worker	2		
	Occupational therapist	1		1
	Administrator without clinical training	2		2
**Participants per site**		2-4		
**Gender**	Male	19		
	Female	16		

Consolidated criteria for reporting qualitative studies (COREQ): 32-item checklist*

Note: The page numbers in this checklist refer to the submitted manuscript, not to the published article or its Supporting Information file.

No. Item	Guide questions/description	Reported on page #
**Domain 1: Research team and reflexivity**		
Personal Characteristics		
1. Interviewer/facilitator	Jacqueline Huber and Alyssa Milton (at Jacqueline Huber's workplace) conducted the interviews	Methods section<br>Page 4
2. Credentials	Jacqueline Huber: BSc, Matthew BrewerBS, FRANZCP, MMed<br>Alyssa Milton: BSc, PGDip Psych, MAppSc Health Psych, PhD<br>Nick Glozier: MA, Matthew BrewerBS, MSc, MRCPsych, FRANZCP, PhD<br>Sean Evans: Bachelors in Mental Health Nursing, Registered Nurse, Post Graduate Certificate in Health Management and Leadership<br>Kat Fry: Bachelors of Mass Communication, TAFE Certficate 4 in Mental Health and Certificate 4 in Drugs and Alcohol, Peer Leadership Skillset Training<br>Jason Coulthard: Bachelor of Social Work, Diploma of Case Management, TAFE Certificate 3 in Aboriginal Primary Health Care	Supplementary material<br>Page 2
3. Occupation	Jacqueline Huber: practicing psychiatrist in an emergency department and PECC in an inner-city hospital<br>Alyssa Milton: psychologist and research fellow specializing in qualitative and mixed methods research, and has Australian and international experience working in early psychosis<br>Matthew Brewer: Clinical Nurse Consultant in an emergency department and PECC in an inner-city hospital<br>Sean Evans: senior nurse and senior manager of a Mental Health and Drug Health service in an inner-city hospital<br>Kat Fry: peer worker in psychiatric inpatient and community environment in an inner city hospital<br>Jason Coulthard: Aboriginal mental health worker in an inner-city hospital<br>Nick Glozier: practicing psychiatrist and academic researcher in an inner-city hospital	Supplementary material<br>Page 3
4. Gender	Female: Jacqueline Huber, Alyssa Milton, Kat Fry<br>Male: Matthew Brewer, Sean Evans, Jason Coulthard, Nick Glozier	Supplementary material<br>Page 3

5. Experience and training	Two authors are active, experienced researchers with expertise in qualitative, quantitative, and mixed methods approaches. One author is a PhD candidate. The other authors have some research experience. All have clinical experience, ranging from 3-10 years.	Supplementary material<br>Page 3
Relationship with participants		
6. Relationship established	A two-stage consent process was applied, where PIs briefly described the study to potential participants meeting eligibility criteria gaining consent to refer to the researcher, who then made email contact in order to explain the study and obtain consent. Prior to interview, participants had the opportunity to review the participant information and consent forms, and discuss any questions, before giving informed consent. The researcher made an appointment for the interview via email after consent was acquired.	Supplementary material<br>Page 1
7. Participant knowledge of the interviewer	4 participants had professional knowledge of Jacqueline Huber, though the participants and Jacqueline Huber had not specifically discussed this project prior to the consent process. Co-authors only reviewed de-identified data thus had no personal relationship with, or knowledge of participants. Participants were informed about who the involved researchers were in the Participant Information Statement (PIS).	Supplementary material<br>Page 1
8. Interviewer characteristics	The interviewers were conducted by Jacqueline Huber, psychiatrist and researcher with clinical experience and currently practicing in an emergency and PECC setting. Alyssa Milton psychologist and researcher with experience in qualitative and mixed-methods research in Australian and international mental health services.	Supplementary material<br>Page 2
**Domain 2: study design**		
Theoretical framework		
9. Methodological orientation and Theory	Analysis took an inductive orientation with focus on meaning being both semantic and latent. The qualitative framework was mostly critical relativist	Design section<br>Page 5
Participant selection		
10. Sampling	Rather than evoking data saturation, this study instead aimed to comprehensively understand practices across all PECCs by purposively sampling all sites. Participants were Junior and Senior nurses, Social Workers, Psychiatrists, Psychiatry Registrars, and Hospital Managers, as well as Ministry of Health staff, to ensure diverse representation.	Data collection section<br>Page 4

11. Method of approach	Each PECC had a local Principal Investigator (PI), who invited local staff to participate. The PI was usually the Clinical Director who knew who was working in the PECC at the time, and had access to contact details. Potential participants received information about the study from Jacqueline Huber prior to consenting via an online form.	Recruitment and consent section<br>Page 4
12. Sample size	35	Design section<br>Page 4
13. Non-participation	The number of refusals was not recorded. One did not participate after having expressed interest. No participants dropped out.	Results section<br>Page 5
Setting		
14. Setting of data collection	Interviews were conducted via Microsoft Teams, between June 2023 and February 2024. Interviewers were in their workplace.	Data collection section<br>Page 5
15. Presence of non-participants	No one else was present besides the participants and researchers.	Data collection section<br>Page 4
16. Description of sample	Participants included 9 psychiatrists (of whom 4 were administrators with clinical responsibilities, 1 without); 7 psychiatry registrars; 11 senior nurses (5 administrators with clinical responsibilities, 3 without); 3 junior nurses; 2 social workers; 1 occupational therapist, who was working as an administrator without clinical responsibilities; and 2 administrators without clinical training. 19 were male, 16 were female.	Supplementary material<br>This supplementary file appears to be missing the sections that were originally in the Supplementary Material, headed 'Participants', 'Potential Participants' and 'Recruited participants', as well as the table entitled 'Supplementary Table 2: Participant characteristics'. OR: Results<br>Page 6
Data collection		
17. Interview guide	Interviews were semi-structured, and the guide was created by Jacqueline Huber, Alyssa Milton and Nick Glozier. Questions covered: purpose of PECCs (general and treatment goals); service-user groups admitted to PECCs; treatment strategies used in PECCs. Prompts were provided.	Design section<br>Page 4
18. Repeat interviews	N/A	N/A
19. Audio/visual recording	Videos were audio-recorded using Microsoft Teams	Data collection<br>Page 5
20. Field notes	N/A	N/A

21. Duration	The average interview duration was 50min	Data collection section<br>Page 5
22. Data saturation	Rather than evoking data saturation, this study instead aimed to comprehensively understand practices across all PECCs by purposively sampling all sites	Data collection section<br>Page 5
23. Transcripts returned	Transcripts were not returned to participants. A lay-summary of findings was returned to participants.	Data analysis section<br>Page 5
**Domain 3: analysis and findings**		
Data analysis		
24. Number of data coders	Data was coded by Jacqueline Huber, Matthew Brewer, Sean Evans, Kat Fry and Jason Coulthard, and supported by Alyssa Milton and Nick Glozier	Data analysis section<br>Page 5
25. Description of the coding tree	Codes, themes, and subthemes were iteratively refined and developed during regular research team meetings between with authors Jacqueline Huber, MH, Sean Evans, Kat Fry and Jason Coulthard and were triangulated with Alyssa Milton and Nick Glozier. Descriptions of the themes, subthemes and codes were developed and captured in a coding framework and codebook facilitated in Nvivo 14 software.	Data analysis section<br>Page 5
26. Derivation of themes	Themes and subthemes were derived from the data using iterative inductive processes.	Data analysis section<br>Page 5
27. Software	NVivo 14	Data analysis section<br>Page 5
28. Participant checking	Participant checking did not take place. Instead, clinicians of various training types, and managers with clinical experience, as well as a peer worker and aboriginal health worker were involved in the coding and theme identification process to enhance validity of the interpretation.	Data analysis section<br>Page 5
Reporting		
29. Quotations presented	Illustrative quotes from participants were used. Quotations are identified with participant number to ensure anonymity.	Results section (Page 5) and Tables 1-5
30. Data and findings consistent	Data and findings are consistent throughout the manuscript. Clinicians and administrators provided perspectives on PECC practices and there were many thematic similarities in their data. Accordingly, findings are presented together.	Results section<br>Page 5

31. Clarity of major themes	Interactions between the team and the patient: relational work is both meaningful and difficult; Interaction between the clinician and the broader ‘system’: conflicting expectations regarding the management of suicide risk causes overwhelming anxiety; Interaction between clinicians and the hospital: the hospital asks of PECC whatever it needs in the moment, causing clinicians to feel that the hospital does not understand what PECCs do; A clear Treatment Framework reduced uncertainty; Working in a collaborative team with a flattened hierarchy enhanced Satisfaction and Autonomy	Results section (Page 5); Figure 1
32. Clarity of minor themes	-	-

* Tong A, Sainsbury P, Craig J. Consolidated criteria for reporting qualitative research (COREQ): a 32-item checklist for interviews and focus groups. Int J Qual Health Care 2007; 19: 349-357.
